# The effects of parent-child relationship, study stress, and mobile phone use on depressive symptoms among Chinese elementary school students: a moderated mediation model

**DOI:** 10.3389/fpsyt.2025.1555120

**Published:** 2025-03-13

**Authors:** Yiting Kong, Zhewei Su, Rui Wang, Jianyu Tan, Yuancen Zhong, Ming Ai, Wo Wang, Su Hong, Qi Zhang, Li Kuang

**Affiliations:** ^1^ Department of Psychiatry, The First Affiliated Hospital of Chongqing Medical University, Chongqing, China; ^2^ Psychiatric Center, The First Affiliated Hospital of Chongqing Medical University, Chongqing, China; ^3^ Mental Health Center, University-Town Hospital of Chongqing Medical University, Chongqing, China

**Keywords:** parent-child relationship, depressive symptoms, study stress, mobile phone use, elementary school students

## Abstract

**Introduction:**

With the rising prevalence of depressive symptoms among younger individuals, depressive symptoms in elementary school students have become a focal issue of concern in society. This study investigated the association between parent-child relationship and depressive symptoms among Chinese elementary school students while testing a moderated mediation model to examine the mediating role of significant study stress and the moderating effect of frequent mobile phone use on this relationship.

**Methods:**

We recruited elementary school students from grades 3 to 6 in S district of Chongqing, China, totaling 33,285 participants (51.72% girls; mean age = 10.36 years, SD = 1.24). Data was analyzed using structural equation modeling to assess the mediating and moderating effects of study stress and mobile phone use, respectively, on the relationship between parent-child relationship and depressive symptoms.

**Results:**

Depressive symptoms prevalence in our study population was 16.3%. Both fair and poor parent-child relationships were significantly linked to an increased risk of depressive symptoms, with study stress serving as a mediator (indirect effects: fair parent-child relationship = 0.058, poor parent-child relationship = 0.031, p < 0.001). Frequent mobile phone use amplified the impact of fair parent-child relationships on study stress (β=0.024, SE=0.016, p < 0.05) and depressive symptoms (β = 0.021, SE = 0.018, p < 0.05) but did not moderate the relationship between poor parent-child relationships and these outcomes.

**Discussion:**

These results emphasize the importance of nurturing parent-child relationship, monitoring study stress, and managing mobile phone usage to support students’ mental health. Furthermore, the findings suggest that the impact of mobile phone usage on the mental health of elementary school students varies in complexity across different parent-child relationship contexts, providing valuable insights and recommendations for developing targeted preventive interventions for depressive symptoms in this demographic.

## 1 Introduction

Depressive symptoms are a debilitating mental health condition, characterized by persistent low mood and associated symptoms such as sleep disturbances and a lack of interest in daily activities (1). Over recent decades, depressive symptoms has shown a trend toward younger populations, impacting an increasing number of children and adolescents. Children in the elementary school stage are psychologically immature and are prone to experiencing depressive emotions and feelings of frustration under stressful conditions (2). In Western populations, the prevalence of depressive symptoms in school-aged children ranges from 9.85% to 10.6% (3–5). However in China, the rate is higher, reaching approximately 17.2% (6), with a rising trend observed in recent years (7). This has raised widespread concern about the mental health of elementary school students across society. If left untreated, childhood or adolescent depressive symptoms can lead to various adverse outcomes in adulthood, including adult psychiatric disorders, substance use problems and functional impairments (8, 9). Therefore, understanding the risk factors and mechanisms of depressive symptoms in elementary school students, and implementing timely interventions, is crucial for their healthy development.

Based on Bowlby’s attachment theory (10), early attachment relationships with primary caregivers (often parents) profoundly impact emotional development and mental health. Secure attachment fosters emotional stability and self-confidence in children, while insecure attachment may lead to feelings of insecurity and emotional problems in childhood (11). The parent-child relationship, as one of the most important relationships during childhood, significantly influences the long-term development of mental health and cognition (12). Specifically, a positive parent-child relationship can serve as a protective factor that reduces internalized symptoms such as depressive symptoms and anxiety symptoms in children (13). Conversely, greater levels of parent-child conflict have been found to be associated with more severe depressive symptoms in children during follow-up studies (14). Similar findings have been observed in studies focusing on Chinese population, longitudinal research among Chinese middle school students indicated that better parent-child relationship correlate with a lower risk of depressive symptoms (15). In a longitudinal study involving Chinese children aged 4-15 found that early parent-child separation had a significant negative impact on children’s depressive symptoms, social skills and academic performance (16). While many studies support the association between parent-child relationship and depressive symptoms, the complex mechanisms underlying these associations remain inadequately elucidated. A recent investigation into the relationship between childhood maltreatment and depressive symptoms, through the construction of a mediation model, has revealed that the parent-child relationship can serve as a mediating factor in the linkage between childhood maltreatment and depressive symptoms (17). This finding provides a novel perspective, suggesting the potential to establish a mediation-modulation model. Existing research has rarely explored mediation-modulation studies with parent-child relationships as the independent variable and depressive symptoms as the dependent variable. By adopting parent-child relationships as the independent variable and depressive symptoms as the dependent variable, it becomes possible to further investigate the association between parent-child relationships and depressive symptoms.

It is important to understand the mediating factors in this relationship. In addition to parent-child relationship, study stress has increasingly become a focal issue for school-aged children as societal competition intensifies (18). Study stress refers to the psychological and physiological tension due to various internal and external factors during academic activities. This stress often arises from concerns about academic performance, time management, and future prospects. Numerous studies have reported that high levels of study stress not only negatively impact students’ academic performance and sleep quality (19), but can also lead to emotional problems such as depressive symptoms (20) and even suicidal behaviors (21). Previous research has indicated that positive parent-child relationship significantly alleviates study stress (22). For elementary school children, where both parent-child relationship and study tasks are crucial, there may be an even closer link between these two factors. For instance, Liao et al. (23) found that parent-child relationship negatively predicts study stress among Chinese elementary school students. Fu et al. (24) found that adolescents’ study stress was positively associated with their depressive symptoms. Considering the close relationship among parent-child relationship, study stress, and depressive symptoms, a study by Qin et al. (25) on Chinese middle school students revealed that the significant negative predictive effect of parent-child relationship on adolescent depressive symptoms can be mediated by poor school adaptation. These research findings provide robust evidence supporting our hypothesis that study stress may mediate the relationship between parent-child relationships and depressive symptoms in elementary school students.

With the development of the internet and the widespread use of mobile phones, the frequency of children’s mobile phone use has been increasing annually. According to the China Internet Network Information Center (26), the number of minor internet users reached 193 million in 2022, and the internet penetration rate of students among elementary school students has risen from 89.5% to 95.1% over the past five years. Based on the uses and gratifications theory (27), unmet needs often drive individuals to use social media, including mobile phones, to fulfill those needs. Previous studies on adolescents have found that those experiencing parental conflict are more likely to overuse mobile phones to compensate for unmet family needs (28), with some studies have even found a significant negative correlation between these relationships and mobile phone addiction (29).

These findings suggest that poor parent-child relationship may be linked to increased mobile phone use, and mobile phone use problems have also been associated with chronic stress to some extent (30). Additionally, research has shown that higher frequency of mobile phone use can lead to more emotional and behavioral problems in both children and adolescents. Twenge et al. (31) found that for children and adolescents, after more than one hour of screen time per day, the longer the screen time, the lower their mental health levels, including diminished self-control and emotional stability. Furthermore, this study found that excessive screen time was linked to a higher likelihood of depressive symptoms or anxiety. Hosokawa et al. (32) found that children’s frequent use of mobile devices was associated with emotional/behavioral problems. Some research focusing on primary and middle school students found that the use of mobile phones can have a negative effect on their mental health (33, 34). Kim et al. (35) also found that self-esteem could have a significant negative effect on mobile phone dependency, and the influence of depression on mobile phone dependency was not significant. Xu et al. (36) found that poor parent-child relationship and problematic mobile phone use were significantly associated with non-suicidal self-injury respectively among elementary school students, underscoring the importance of concurrently investigating both parent-child relationships and mobile phone use.

The aforementioned studies collectively indicate that the frequency of mobile phone use may be closely related to parent-child relationship, stress, and depressive symptoms among elementary school students. However, despite the growing recognition of the importance of these factors, prior research has largely overlooked the combined mediating and moderating roles of study stress and mobile phone use in the relationship between parent-child relationships and depressive symptoms.

Moreover, due to restrictions related to the protection of minors’ privacy, obtaining informed consent and support from parents, schools, and other stakeholders to collect large-scale mental health data from elementary school students in China faces significant challenges. As a result, the number of studies on depressive symptoms in elementary school students is relatively limited, with most sample sizes being under 4,000 and the largest around 6,000 participants (6). Additionally, while previous research has examined the relationship between parent-child relationship and depressive symptoms, these studies have typically treated the parent-child relationship as a continuous variable, without categorizing it by degree for a more nuanced understanding of its impact.

**Figure 1:**
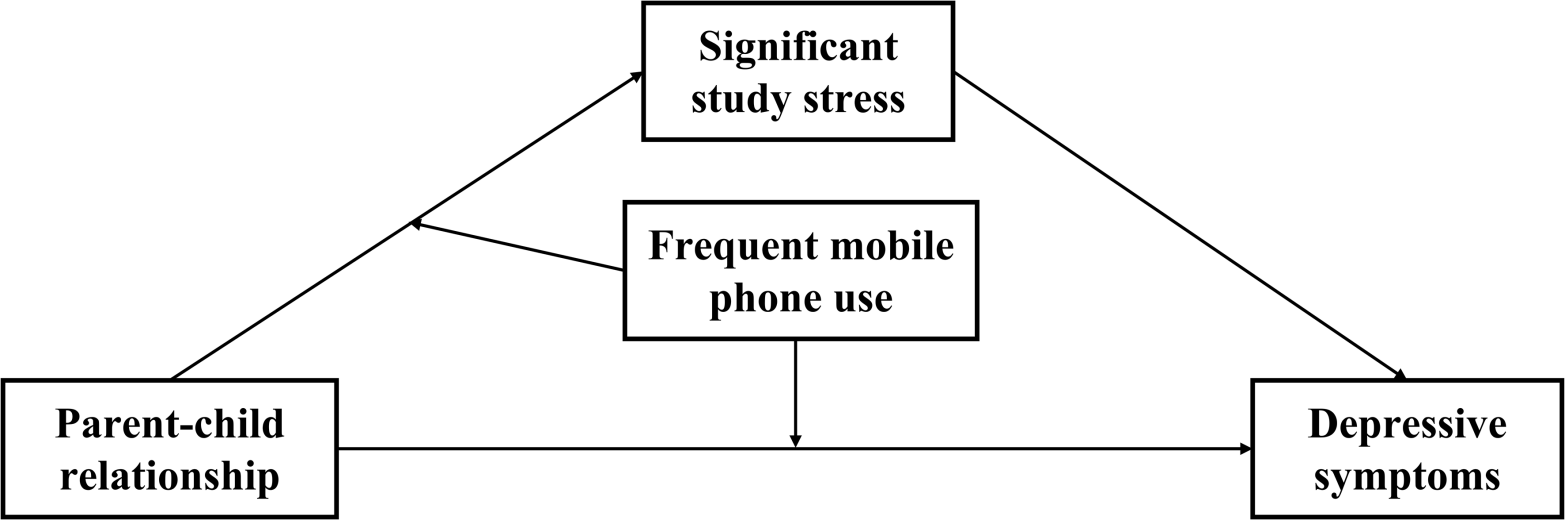
Conceptual model.

To address these gaps, this study was designed as a cross-sectional survey, collecting questionnaire data from 33,285 elementary school students in grades 3-6 from a district in Chongqing. As illustrated in **Figure 1**, we constructed a hypothesized moderated mediation model to investigate the relationship between varying degrees of parent-child relationship and depressive symptoms in elementary school students. Specifically, we focused on the mediating role of study stress and the moderating effect of frequent phone use. By using categorical variables to examine different levels of parent-child relationship quality, this study aims to provide a more nuanced understanding of how these factors interact to influence depressive symptoms. The findings are expected to offer valuable insights for improving prevention and intervention strategies for depressive symptoms in elementary school students.

Hypothesis 1: Compared to good parent-child relationship, both fair and poor parent-child relationships are positively correlated with depressive symptoms.
Hypothesis 2: Significant study stress mediates the relationship between fair/poor parent-child relationships and depressive symptoms, with the indirect effect being stronger for poor parent-child relationship than for fair one.
Hypothesis 3a: Frequent mobile phone use moderates the direct relationship between fair parent-child relationships and depressive symptoms via significant study stress.
Hypothesis 3b: Frequent mobile phone use moderates the direct relationship between poor parent-child relationships and depressive symptoms via significant study stress.

## 2 Materials and methods

### 2.1 Participants

Participants were 3rd to 6th graders from elementary schools located in District S of Chongqing, China. We used cluster sampling to select all schools and classrooms within this region for the study. Considering the suitability of the questionnaire for different age groups and the ability to fully comprehend the questions, 1st and 2nd grade students were not included in the study. Written informed consent was obtained from all participants and their guardians. After excluding those who did not complete the entire questionnaire, a total of 33,285 students participated in the study (51.72% girls, n = 17214; mean age = 10.36 years, SD = 1.24).

### 2.2 Measures

Given the young age of the participants and the cultural context of the study, shorter and less intrusive tools were selected to minimize participant fatigue and discomfort. These tools were pilot-tested and shown to be reliable and valid for this age group and setting.

#### 2.2.1 Depressive symptoms

Depressive symptoms were assessed using the Children’s Depression Inventory (CDI) Questionnaire, which is suitable for children and adolescents aged 7 to 17 (37). The CDI scale comprised 27 items, covering five subscales: anhedonia, negative mood, low self-esteem, ineffectiveness, and interpersonal problems. To control for the effect of default responses, 14 items were positively scored, and 13 items were negatively scored. Each item consisted of three similarly structured sentences (e.g., “I occasionally feel tired,” “I often feel tired,” “I always feel tired”). Items were graded on a scale from 0 to 2, where a score of 0 indicates a general reaction, a score of 1 indicates a moderate presence of depressive symptom, and a score of 2 indicates a pronounced presence of depressive symptom, with a maximum total score of 54 points. A higher total score indicates greater severity of depressive symptoms, with a total score ≥ 19 indicating meaningful depressive symptoms (38).

In this study, to better explore the differences between participants with and without depressive symptoms, we categorized participants based on their CDI total scores. Participants with a CDI score ≥ 19 were defined as the group with significant depressive symptoms and assigned a value of 1, while those with a CDI score < 19 were defined as the group without significant depressive symptoms and assigned a value of 0. In a study by Wu et al. (39) Cronbach’s alpha for the CDI scale was reported to be 0.88 in Chinese elementary and middle school students. In our study, the Cronbach’s alpha for the CDI was 0.884, indicating a good internal consistency.

#### 2.2.2 Parent-child relationship

The study incorporated several questions in the Basic Information Questionnaire to collect the basic information of students. Participants responded to the question, “How is your relationship with your parents?” with options: 1=Good, 2=Fair, 3=Poor. The Parent-Child relationship was assessed based on the answers to this question, which categorizes parent-child relationships into three distinct levels: good, fair, and poor. This approach aligns with developmental theories, such as Bowlby’s attachment theory (40), and helps explore how varying relationship dynamics impact depressive symptoms, offering a more nuanced understanding.

#### 2.2.3 Study stress

The General Condition Scale included the question, “Do you feel stressed about your studies?” with response options: “Not significant,” “Moderate”, and “Significant” (0=Not significant, 1=Moderate, 2= Significant). This categorization of study stress into low, moderate, and high levels reflects how students typically perceive academic pressure, such as homework load and examination schedules. By classifying stress in this way, the study aims to explore its differential impact on depressive symptoms, providing a more nuanced analysis that aligns with real-world educational contexts.

#### 2.2.4 Frequency of mobile phone use

The General Condition Scale also collected, “How often do you use the internet or your phone?” with response options: “Rarely,” “Sometimes”, and “Often” (1=Rarely, 2=Sometimes, 3=Often). In this study, students who selected “Often” for this question were considered to have a habit of frequent mobile phone use.

### 2.3 Procedure

The study was based on the mental health screening data of elementary school students from District S in Chongqing, collected between September and December 2022, and the survey was paused during school exams and holidays. With the cooperation of school authorities, all 3rd to 6th grade students were invited to participate in the survey, with informed consent obtained from parents, teachers and students. The participating students spent approximately 30 minutes of their computer class time completing the online self-report questionnaires, under the supervision of teachers trained in using these questionnaires. Data collection was carried out by trained mental health teachers and graduate students majoring in psychology. To encourage honest responses, participants were informed that their participation was entirely voluntary so that they could submit or withdraw their responses at any time, and their personal information and responses would be kept strictly confidential.

Besides, students’ mental health risks were classified as high or low. High-risk students received psychiatric interviews to determine warning levels (1 to 4), guiding specific interventions, such as immediate care and safety monitoring for level 1. This ensured timely mental health support for participants.

### 2.4 Statistical analyses

Given that the variables in this study are ordinal variables, we used case numbers and proportions to describe them, with Spearman correlation analysis applied to observe relationships among variables. First, data preprocessing, descriptive statistics, and correlation analysis were conducted using SPSS software version 26.0. Second, as the study focuses on the relationships between fair parent-child relationship, poor parent-child relationship, significant study stress, frequent mobile phone use, and depressive symptoms, variables were dummy-coded (41) for further analysis. Specifically, fair parent-child relationship, poor parent-child relationship, significant study stress, and frequent mobile phone use were coded as 1, with all other response options assigned a value of 0. Structural equation modeling (SEM) (42) was then performed using Mplus Version 8.1, applying maximum likelihood estimation and bias-corrected percentile bootstrapping (5,000 iterations). This analysis aimed to examine whether significant study stress mediates the relationship between fair/poor parent-child relationship and depressive symptoms. Following this, the study assessed whether frequent mobile phone use moderated both the direct and indirect pathways and calculated the relevant effects. For handling missing data, we used listwise deletion, which involves removing cases with missing values from the analysis. This method was chosen based on its robustness and its minimal impact on the integrity of the results. Gender (Boys/Girls) and age were controlled for in the regression equations as part of the SEM process. This study reported the 95% confidence intervals (CI) for the relevant effects. The effect sizes were primarily represented by standardized regression coefficients (β values) in the text, which were used to assess the strength of the relationships between variables.

### 2.5 Ethics statement

Ethical approval for the study was obtained from the Ethics Committee of the First Affiliated Hospital of Chongqing Medical University (registration number: 2020-879, December 16th, 2020).

## 3 Results

### 3.1 Descriptive statistics

**Table 1** presents the demographic statistics of the participants. **Table 2** presents the frequency, proportion, and correlations for the measured variables. First, the detection rate of depressive symptoms in the study population was found to be 16.3%. Then, as expected, parent-child relationship, significant study stress, frequent mobile phone use and depressive symptoms were significantly and positively correlated with each other.

**Table 1:** Demographic statistics of the participants.

Variables	N/Mean	%/Standard
Age	10.36	1.24
Gender		
Boys	16071	48.28
Girls	17214	51.72
Depressive symptoms		
Without significant depressive symptoms	27854	83.68
With significant depressive symptoms	5431	16.32
Parent-Child relationship		
Poor	27698	83.21
Fair	5261	15.81
Good	326	0.98
Study stress		
Significant	3174	9.54
Moderate	16876	50.70
Not significant	13235	39.76
Frequency of mobile phone use		
Rarely	12133	36.45
Sometimes	17047	51.22
Often	4105	12.33

**Table 2:** Descriptive statistics and correlations of the study variables.

	1	2	3	4	5
1. Depressive symptoms	−				
2. Significant study stress	0.338^**^	−			
3. Parent-child relationship = 2	0.305^**^	0.209^**^	−		
4. Parent-child relationship = 3	0.139^**^	0.104^**^	-0.043^**^	−	
5. Frequent mobile phone use	0.202^**^	0.159^**^	0.155^**^	0.066^**^	−
Frequency	5431	3174	5261	326	4105
Proportion (%)	16.3	9.5	15.8	1.0	12.3

^**^ *p*<0.01.

### 3.2 Testing for mediation effect

**Figure 2:**
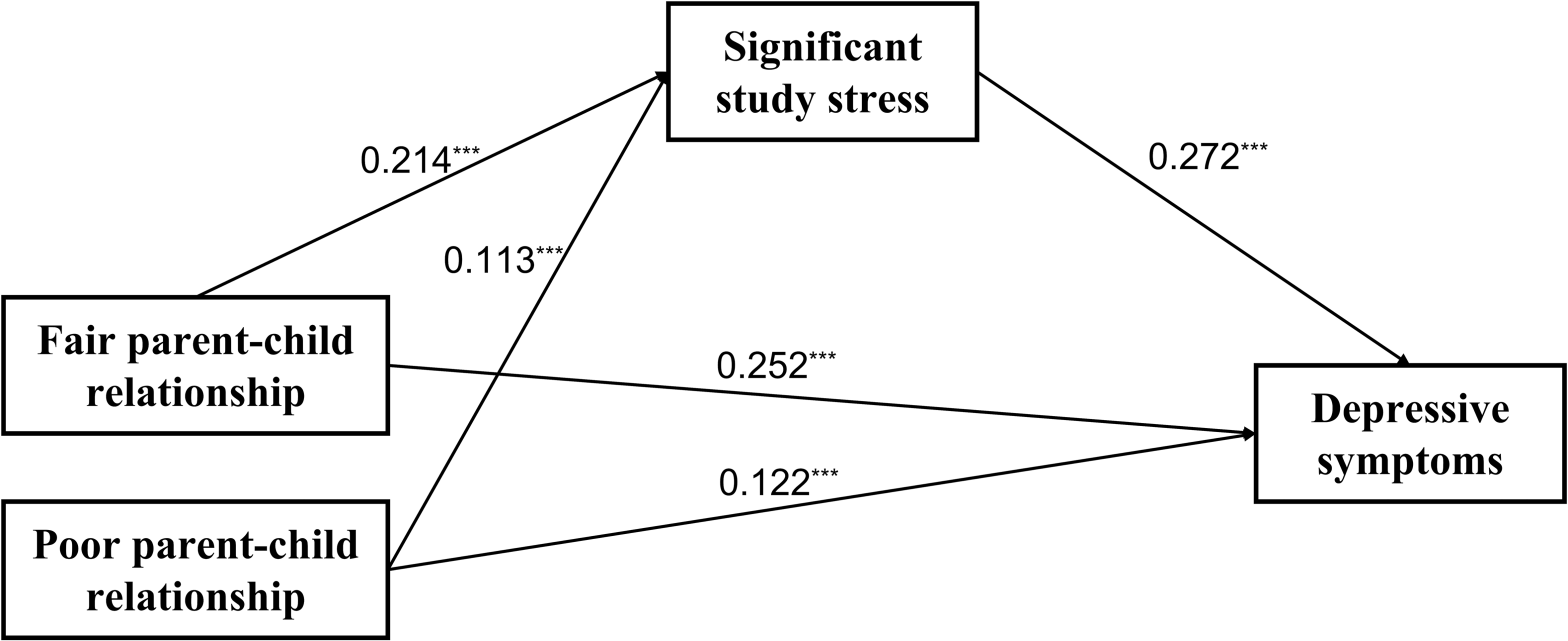
The mediating effect of significant study stress in the association between parent-child relationships and depressive symptoms. ^***^ *p* < 0.001.

**Figure 2** illustrates the mediating role of significant study stress in the relationship between parent-child relationships and depressive symptoms. A mediation effect is considered significant when the confidence interval (CI) excludes 0. As shown in **Table 3**, after adjusting for gender and age, a fair parent-child relationship was found to be positively associated with significant study stress (β = 0.214, SE = 0.007, t = 29.922, p < 0.001) and depressive symptoms (β = 0.122, SE = 0.007, t = 17.120, p < 0.001). Similarly, a poor parent-child relationship was also positively associated with both significant study stress (β = 0.113, SE = 0.010, t = 11.829, p < 0.001) and depressive symptoms (β = 0.272, SE = 0.007, t = 37.597, p < 0.001). Furthermore, significant study stress was positively correlated with depressive symptoms (β = 0.252, SE = 0.007, t = 37.137, p < 0.001).

**Table 3:** The mediating role of significant study stress.

Outcome variable	Predictor	Estimate	SE	t-value	p-value
Depressive symptoms	Significant study stress	0.272^***^	0.007	37.597	<0.001
	X1	0.252^**^	0.007	37.137	<0.001
	X2	0.122^***^	0.007	17.120	<0.001
Significant study stress	X1	0.214^***^	0.007	29.922	<0.001
	X2	0.113^***^	0.010	11.829	<0.001

After adjusting for gender and age.

X1: fair parent-child relationship. X2: poor parent-child relationship.

^**^ *p*<0.01,^***^ *p*<0.001.

The bias-corrected bootstrapping analysis confirmed that significant study stress partially mediated the relationship between parent-child relationships and depressive symptoms. As shown in **Table 4**, for a fair parent-child relationship, the indirect effect was 0.058 (t = 22.602, CI = [0.05, 0.06]), while for a poor parent-child relationship, the indirect effect was 0.031 (t = 11.248, CI = [0.03, 0.04]). The mediation effect accounted for 19% of the total effect for the fair parent-child relationship (β = 0.310, t = 45.616, p < 0.001, CI = [0.30, 0.32]) and 20% of the total effect for the poor parent-child relationship (β = 0.153, t = 19.105, p < 0.001, CI = [0.14, 0.17]).

**Table 4:** The mediation effects in direct and indirect effects of the model.

Mediating variable	Effect	Effect size	95%CI	t-value	Effect ratio
From X1 to Y	Total effect	0.310^***^	(0.30, 0.32)	45.616	
	Direct effect	0.252^***^	(0.24, 0.27)	37.137	
	Indirect effect	0.058^***^	(0.05, 0.06)	22.602	19%
From X2 to Y	Total effect	0.153^***^	(0.14, 0.17)	19.105	
	Direct effect	0.122^***^	(0.11, 0.14)	17.120	
	Indirect effect	0.031^***^	(0.03, 0.04)	11.248	20%

After adjusting for gender and age.

X1: fair parent-child relationship. X2: poor parent-child relationship. Y: depressive symptoms.

^**^ *p*<0.01,^***^ *p*<0.001.

### 3.3 Testing for moderated mediation effect

**Figure 3:**
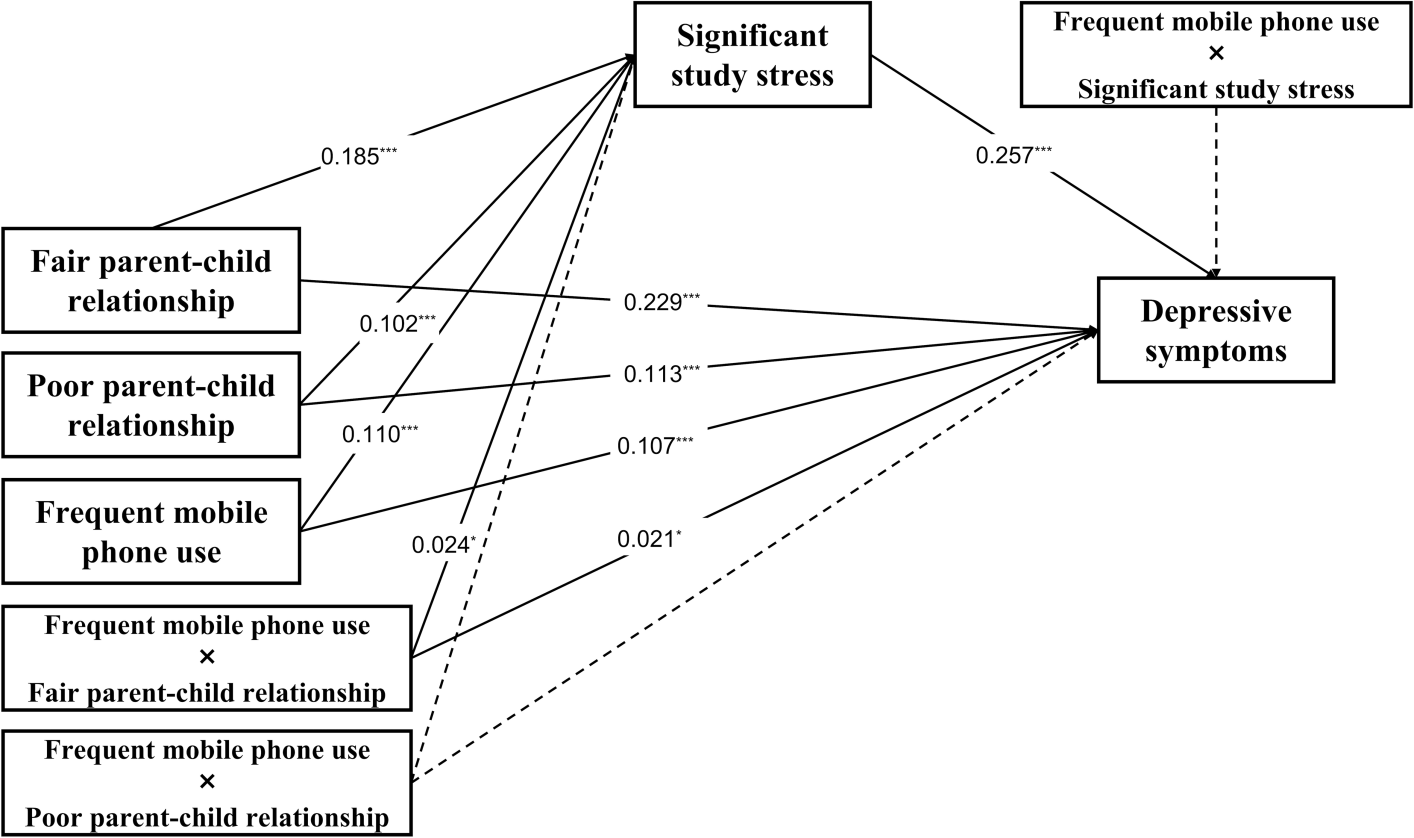
The moderating effect of frequent mobile phone use on the direct and indirect associations between parent-child relationships and depressive symptoms. ^*^ *p*<0.05, ^***^ *p* < 0.001.

Then, we tested the moderated mediation effect of frequent mobile phone use in **Figure 3**. The results demonstrated that fair parent-child relationship (β=0.229, SE=0.008, p<0.001), poor parent-child relationship (β=0.113, SE=0.030, p<0.001), significant study stress (β=0.257, SE=0.009, p<0.001) and frequent mobile phone use (β=0.107, SE=0.008, p<0.001) were all significantly associated with depressive symptoms. Additionally, fair parent-child relationship (β=0.185, SE=0.007, p<0.001), poor parent-child relationship (β=0.102, SE=0.033, p<0.001) and frequent mobile phone use (β=0.110, SE=0.007, p<0.001) were significantly associated with significant study stress.

Crucially, frequent mobile phone use significantly moderated the impacts of fair parent-child relationship on both significant study stress (β=0.024, SE=0.016, p=0.026) and depressive symptoms (β=0.021, SE=0.018, p=0.021), which indicates that frequent mobile phone use can increase the significant study stress of elementary school students with fair parent-child relationship. However, the impact of poor parent-child relationship on significant study stress and on depressive symptoms were not significantly moderated by frequent mobile phone use (Further details can be found in **Supplementary Table 1**).

**Figure 4:**
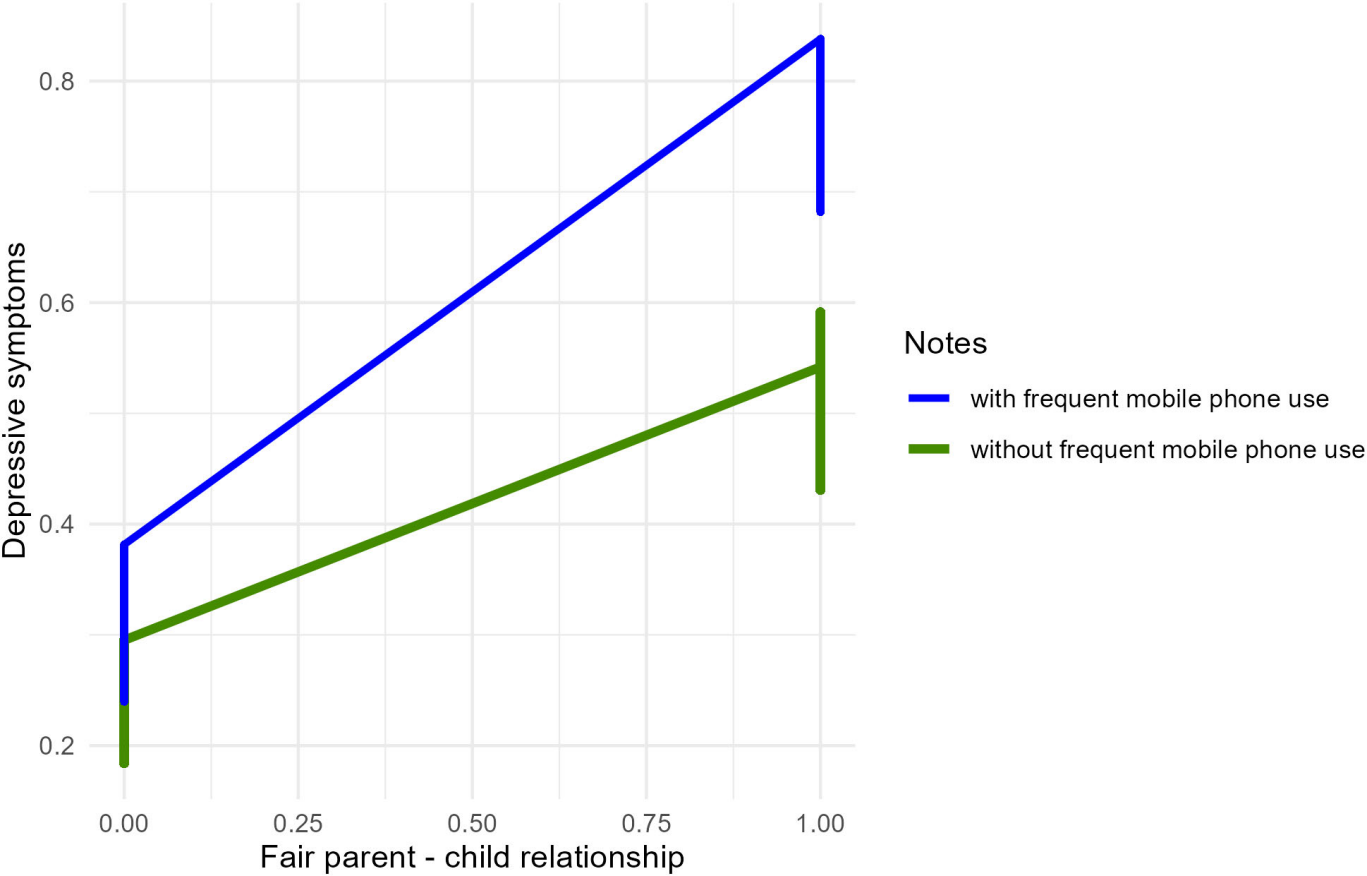
Moderation interaction plot.

Finally, we further estimated the direct and indirect effects of the model. As shown in **Table 5**, the indirect effect of significant study stress was higher on fair parent-child relationship (indirect effect=0.060, SE=0.005, t=11.762, p<0.001), than those without the moderator (indirect effect=0.048, SE=0.003, t=19.006, p<0.001). Similarly, the direct effect between fair parent-child relationship and depressive symptoms (direct effect=0.272, SE=0.016, t=17.145, p<0.001) was also higher compared with those without being moderated (direct effect=0.232, SE=0.008, t=30.419, p<0.001). Therefore, the mediating effect of significant study stress in the association between fair parent-child relationship and depressive symptoms was moderated by frequent mobile phone use. **Figure 4** presents the moderation interaction plot, visually illustrating these moderation effects.

**Table 5:** The moderation effects in direct and indirect effects of the model.

Variable	Estimate	SE	t-value	p-value
X1 to M_NW	0.048^***^	0.003	19.006	<0.001
X1 to M_WW	0.060^***^	0.005	11.762	<0.001
X1 to Y_NW	0.232^***^	0.008	30.419	<0.001
X1 to Y_WW	0.272^***^	0.016	17.145	<0.001
TOTAL_NW	0.280^***^	0.008	35.487	<0.001
TOTAL_WW	0.332^***^	0.016	20.146	<0.001

After adjusting for gender and age.

X1: fair parent-child relationship. Y: depressive symptoms. M: significant study stress. NW: without the moderator. WW: with the moderator. TOTAL: total effect from X1 to Y.

^***^ *p*<0.001.

## 4 Discussion

Depressive symptoms in younger populations is increasingly becoming a critical public health concern. This study explores the impact of parent-child relationships on depressive symptoms in elementary school students, with a focus on the mediating role of study stress and the moderating effect of mobile phone use. The key findings indicate that study stress mediates the relationship between fair/poor parent-child relationships and depressive symptoms, and frequent mobile phone use exacerbates the negative effects of fair parent-child relationships on both study stress and depressive symptoms.

First, the study found that the depressive symptoms screening rate among 3rd to 6th-grade elementary school students was 16.3%, notably exceeding the global depressive symptoms prevalence rates of 9.85%-10.6% for children and adolescents (3–5). This difference underscores regional variability in depressive symptoms rates, potentially influenced by social, educational, and cultural factors, such as the greater academic stress faced by Chinese students (43). Interestingly, the rate was slightly lower than the previously reported 17.2% prevalence among Chinese elementary school students (6). This discrepancy may stem from differences in sample size. Previous meta-analyses on depressive symptoms in this age group had typically included fewer than 4,000 participants (6), whereas this study’s sample size exceeded 30,000, possibly contributing to the marginally lower detection rate.

Second, our findings were consistent with Hypothesis 1, indicating that both fair and poor parent-child relationships are significantly associated with depressive symptoms among elementary school students compared to good parent-child relationship. From birth, individuals develop deep and lasting bonds with their parents through continuous interactions—what is known as parent-child attachment (44). Classic attachment theory suggests that attachment with parents influences children’s internal working models of self and others: secure attachment leads children to view themselves as valuable and others as trustworthy, while insecure attachment fosters negative internal working models, resulting in negative self-representations and perceptions of others. This, in turn, leads to a range of negative emotional, cognitive, and behavioral patterns, causing depressive symptoms in children (45, 46). This explains why children with weaker parent-child relationship are more susceptible to depressive symptoms. Compared to good parent-child relationship, fair parent-child relationship provides insufficient support, when parental counseling, guidance, and advice are less appropriate or specific, children’s susceptibility to stressors may increase, thereby elevating the likelihood of developing depressive symptoms (47). Harsh and indirect parenting styles may also distinguish good parent-child relationship from fair one, and such parenting practices are associated with an increased risk of depressive symptoms in adolescents (48). Our findings are consistent with previous studies both domestically and internationally (46, 49), which has consistently shown a strong correlation between parent-child relationship quality and childhood depressive symptoms. However, past studies have often treated the parent-child relationship as a continuous variable, finding that poorer relationships are associated with more severe depressive symptoms in children. Our study not only reaffirms the critical role of parent-child relationship in children’s healthy development but also reveals that both fair and poor parent-child relationships can act as risk factors significantly associated with depressive symptoms in elementary school students. Additionally, conducted during the COVID-19 pandemic revealed that family isolation policies significantly increased the time Chinese elementary school students spent with their parents, making the parent-child relationship a primary source of interpersonal support. As a result, the influence of parents on their children’s emotional development has increased in China (2).

Third, the study verified the mediating role of significant study stress between parent-child relationship and depressive symptoms among elementary school students. Specifically, the proportion of indirect effect of study stress between fair parent-child relationship and depressive symptoms is 19%, while it is 20% between poor parent-child relationship and depressive symptoms. Such results are inconsistent with Hypothesis 2. The discrepancy may be attributed to the possibility that the relationship between parent-child relationship and study stress is not strictly linear. While poor relationships are typically associated with stronger negative effects, fair relationships may still exert a significant influence due to their inherent instability and partial fulfillment of emotional needs. And the measurement tools used to assess parent-child relationship may not fully capture the nuanced differences between fair and poor relationships, leading to similar effect sizes. According to Beck’s cognitive theory of depressive symptoms (50), the core of the theory lies in maladaptive self-schemas, or generalized self-perceptions formed based on past experiences. These schemas—centered on feelings of failure, worthlessness, and dysfunctional attitudes—constitute cognitive vulnerability to depressive symptoms. Based on this theory, adverse childhood experiences diminish coping abilities as children grow, leading to the formation of early maladaptive self-schemas (51). Previous evidence has also shown that children with insecure attachments display reduced early coping abilities (52). Specifically, children with strong parent-child relationship benefit from enhanced coping abilities and family support, which strengthens their resilience against academic challenges, thereby reducing perceived study stress (53). In contrast, children with poor parent-child relationship may experience greater study stress when facing school challenges, consistent with previous findings (15). These children, when under significant academic burden, might activate maladaptive schemas, leading to negative cognitions about the world, the future, or themselves, ultimately resulting in depressive symptoms.

In contemporary Chinese society, elementary school students face increasing study stress, with Chinese parents often placing high expectations on their children’s academic performance (18). Children frequently internalize these parental expectations to gain parental recognition (54). In families with not good parent-child relationship, children may strive to achieve better academic results to gain parental approval and attention, thus setting higher standards for their academic performance. This intense study stress may lead them to employ more maladaptive coping strategies, resulting in emotional problems (55, 56). Our findings align with those of Liao et al. (2), who reported that not good parent-child relationship influence the development of depressive symptoms in children through significant study stress, emphasizing that good parent-child relationships and family support can help elementary school students manage study stress, preventing or alleviating depressive symptoms. Compared to previous studies, our study separately examined varying degrees of parent-child relationship, finding that significant study stress mediates the impact of both fair and poor parent-child relationships on depressive symptoms among elementary school students. Additionally, our findings suggest that the proportion of the indirect effect of significant study stress on the relationship between both fair and poor parent-child relationships and depressive symptoms is similar. This indicates that attention should be given not only to students with poor parent-child relationship but also to those with fair parent-child relationship, as fluctuations in study stress can also negatively impact their mental health.

Last, the study revealed significant differences in the moderating effects of frequent mobile phone use across different parent-child relationships among primary school students. Specifically, the more frequently children use mobile phones, the stronger the influence of fair parent-child relationship on significant study stress and depressive symptoms. According to the uses and gratifications theory (27) and prior research findings (57), children and adolescents often turn to digital activities, including mobile phone use, to escape real-life stress. However, this behavior can lead to social withdrawal and potentially result in various health issues, including depressive symptoms (58, 59). Previous studies have indicated that frequent mobile phone use as a means of avoiding reality is linked to declining academic performance (60, 61). According to Beck’s cognitive theory (50), this phenomenon may further increase children’s subjective study stress, activating maladaptive schemas that can lead to mental health issues such as depressive symptoms. This finding supports the Hypothesis 3a, suggesting that, for students with fair parent-child relationship, reducing mobile phone use and encouraging real-world activities may help alleviate study stress and lower the risk of depressive symptoms. However, this study did not find that frequent mobile phone use moderated the impact of poor parent-child relationship on study stress and depressive symptoms, as hypothesized in Hypothesis 3b. This discrepancy may be due to the following factors: First, it may stem from differences in sample characteristics. This study focused on Chinese elementary school students, whose experiences may differ from those of populations of different ages, regions, or cultural backgrounds. Second, poor parent-child relationship may exert such a strong direct impact on depressive symptoms that the additional influence of mobile phone use becomes relatively negligible. The study by Song et al. (17) found that parent-child relationships can serve as a mediating factor in the relationship between childhood maltreatment and depressive symptoms, the mediating effect of poor parent-child relationships may attenuate the moderating role of mobile phone use. In other words, the negative effects of poor relationships may overshadow the potential moderating role of mobile phone use. Third, students with poor parent-child relationships may rely less on mobile phones as a coping mechanism compared to those with moderate relationships. Instead, they may turn to other behaviors or strategies to manage stress, such as non-suicidal self-injury (36), which could explain the lack of a significant moderating effect. Finally, the measurement tool used to assess mobile phone use may not fully capture the nuanced ways in which students with poor parent-child relationships interact with technology, potentially leading to an underestimation of its moderating role.

One of the key strengths of this study is its large sample size, which surpasses that of previous research (6). Based on psychological health screening data from S District in Chongqing, the study successfully obtained informed consent and cooperation from government education departments, schools, families, and participants, ultimately including 33,285 students. This substantial sample size enhances the representativeness and reliability of the findings. Moreover, to our knowledge, this study is the first to categorize parent-child relationships into fair and poor classifications for analysis, providing a new perspective on the topic. The results further reveal that frequent mobile phone use exacerbates the impact of fair parent-child relationship on study stress and depressive symptoms. However, it does not moderate the relationship between poor parent-child relationship and study stress or depressive symptoms. This diverges from Hypothesis 3b, highlighting how excessive mobile phone use amplifies the psychological vulnerabilities associated with family environments characterized by fair parent-child relationships. Additionally, the findings also shed light on the complex mechanisms by which frequent mobile phone use influences depressive symptoms in elementary school students, depending on the quality of their parent-child relationship.

The results suggest that improving parent-child relationship may play a crucial role in alleviating study stress and preventing depressive symptoms among elementary school students. Additionally, it highlights that students with both fair and poor parent-child relationships may be highly sensitive to study stress, necessitating equal attention to changes in study stress and the associated risk of depressive symptoms in both groups. Moreover, among students with fair parent-child relationship, frequent mobile phone use may exacerbate their study stress and depressive symptoms. However, the study did not find a moderating effect of mobile phone use frequency on depressive symptoms among students with poor parent-child relationship, indicating that the impact of mobile phone use on the mental health of elementary school students is complex and varied, potentially involving different mechanisms.

The findings of this study hold significant clinical implications for the prevention and intervention of mental health issues among elementary school students. Firstly, the results highlight the critical role of improving parent-child relationships in alleviating study stress and preventing depressive symptoms in children. This suggests that clinical interventions aimed at enhancing parent-child interactions, such as family therapy or parent training programs, could be effective strategies for reducing stress and depressive symptoms in this population. Secondly, the study underscores that students may exhibit high sensitivity to study stress regardless of the quality of their parent-child relationships. This indicates that clinicians and educators should pay equal attention to monitoring and addressing study stress and associated depressive risks in both groups of students—those with positive parent-child relationships and those with strained relationships. Interventions such as stress management workshops or school-based counseling services could be beneficial for all students. Thirdly, the finding that frequent mobile phone use may exacerbate study stress and depressive symptoms in students with fair parent-child relationships suggests that clinicians should consider addressing screen time and digital habits as part of comprehensive mental health interventions. However, the absence of a moderating effect of mobile phone use on depressive symptoms in students with poor parent-child relationships implies that the impact of mobile phone use on mental health is multifaceted and may involve different underlying mechanisms. This complexity calls for tailored interventions that consider individual differences in family dynamics and digital behavior. In summary, these findings advocate for a holistic approach to mental health care that integrates family dynamics, study stress management, and digital behavior regulation. Clinicians and educators should collaborate to develop targeted interventions that address these interrelated factors to promote the psychological well-being of elementary school students.

This study also has several limitations that future research should address. First, the reliance on self-reported data may introduce recall bias. Moreover, the use of single-item measures to assess parent-child relationships and study stress presents several limitations, such as potential measurement errors, reduced validity, and limited discriminatory power. Future research should consider employing more comprehensive, multi-item measures and structured interviews to capture the complexity of these constructs more accurately and explore their interrelationships in greater depth. Additionally, the sample was restricted to elementary school students in Chongqing, China, limiting the generalizability of the findings across diverse age groups and regional contexts. Chongqing, as a major urban center in southwestern China, has unique cultural and socioeconomic characteristics that may not fully represent other regions, particularly rural areas or regions with different educational systems and family dynamics. The emphasis on academic achievement and the competitive educational environment in urban Chongqing may amplify the observed effects of academic stress and parent-child relationships on depressive symptoms. And participants may reflect the socioeconomic status and parenting practices typical of urban families, which may differ from those in rural or less developed areas. Comparative studies across varied age cohorts and cultural backgrounds are warranted to elucidate potential similarities and differences with the current results. Notably, while the large sample size enhanced statistical power, it may also amplify the detection of statistically significant effects with limited practical relevance. For instance, variables with small effect sizes (e.g., ΔR² < 0.01) reached significance due to the sample size but may not translate to meaningful clinical or educational interventions. Future studies should complement statistical significance with effect size benchmarks (e.g., Cohen’s guidelines) to better evaluate the practical implications of findings. Finally, as this is a cross-sectional study, causal relationships among variables remain constrained. Longitudinal research designs are recommended to clarify the causal pathways underlying these relationships. Addressing these limitations in future studies will enable a more nuanced understanding of the dynamic interactions among parent-child relationships, study stress, mobile phone use, and depressive symptoms.

## 5 Conclusion

This study underscores the intricate relationships among parent-child dynamics, study stress, and mobile phone use in influencing depressive symptoms among elementary school students. The findings highlight that even moderate parent-child relationships can contribute to heightened emotional distress, particularly when coupled with excessive mobile phone use as a coping mechanism. Furthermore, study stress emerges as a significant mediator, reinforcing the urgency of addressing academic pressures in tandem with familial interventions. Theoretical contributions extend family systems theory by incorporating digital behavior as a contextual moderator, while practical implications emphasize the necessity of tailored interventions, including parent training, screen-time regulations, and stress-management programs. Despite limitations related to cross-sectional design and sample geographical constraints, this research provides a foundation for future longitudinal investigations into the evolving role of family and technology in child mental health. A holistic, multi-stakeholder approach is essential to mitigating depressive symptoms and fostering emotional resilience in young students.

## Data Availability

The raw data supporting the conclusions of this article will be made available by the authors, without undue reservation.
