# Supplementary material for: The effects of parent-child relationship, study stress, and mobile phone use on depressive symptoms among Chinese elementary school students: a moderated mediation model
Source: Front Psychiatry. 2025 Mar 13;16:1555120. doi: 10.3389/fpsyt.2025.1555120 (PMC11965941; doi:10.3389/fpsyt.2025.1555120)
Supplement: Supplementary file 1 [file Table1.docx]

***Supplementary Table***

***The effects of parent-child relationship, study stress, and mobile phone use on depressive symptoms in Chinese elementary school students: A moderated mediation model***

**Supplementary Table 1** The moderated-mediating effect of significant study stress on depressive symptoms

| Outcome variable | Predictor | Estimate | SE | p-value |  |
| --- | --- | --- | --- | --- | --- |
| Depressive symptoms | Gender | -0.039^***^ | 0.004 | <0.001 |  |
|  | Age | -0.001 | 0.001 | 0.908 |  |
|  | X1 | 0.229^***^ | 0.008 | <0.001 |  |
|  | X2 | 0.113^***^ | 0.030 | <0.001 |  |
|  | W | 0.107^***^ | 0.008 | <0.001 |  |
|  | X1 × W | 0.021^*^ | 0.018 | <0.05 |  |
|  | X2 × W | 0.005 | 0.045 | 0.449 |  |
|  | Significant study stress | 0.257^***^ | 0.009 | <0.001 |  |
| Significant study stress | X1 | 0.185^***^ | 0.007 | <0.001 |  |
|  | X2 | 0.102^***^ | 0.033 | <0.001 |  |
|  | W | 0.110^***^ | 0.007 | <0.001 |  |
|  | X1 × W | 0.024^*^ | 0.016 | <0.05 |  |
|  | X2 × W | 0.006 | 0.058 | 0.609 |  |
| *After adjusting for gender and age*  *Note.* X1: fair parent-child relationship. X2: poor parent-child relationship.W:frequent mobile phone use.  ^*^*p*<0.05,^***^*p*<0.001 | | | | | |
